# Intrafamilial oocyte donation in classic galactosemia: ethical and societal aspects

**DOI:** 10.1007/s10545-018-0179-y

**Published:** 2018-04-18

**Authors:** M. Haskovic, W. J. Poot, R. J. T. van Golde, S. H. Benneheij, E. Oussoren, G. M. W. R. de Wert, A. Krumeich, M. Estela Rubio-Gozalbo

**Affiliations:** 1 Department of Pediatrics and Department of Clinical Genetics, Maastricht University Medical Center+, P. Debeylaan 25, P.O. Box 5800, 6202 AZ Maastricht, The Netherlands; 2 Department of Obstetrics & Gynecology, Maastricht University Medical Center+, Maastricht, The Netherlands; 3 GROW—School for Oncology and Developmental Biology, Maastricht University, Maastricht, The Netherlands; 4 Department of Obstetrics & Gynecology, Division of Reproductive Medicine, Erasmus Medical Center, Rotterdam, The Netherlands; 5 Department of Pediatrics, Erasmus Medical Center, Sophia Children’s Hospital, Rotterdam, The Netherlands; 6 Department of Health, Ethics & Society, Maastricht University, Maastricht, The Netherlands

## Abstract

Classic galactosemia is a rare inherited disorder of galactose metabolism. Primary ovarian insufficiency (POI) with subfertility affects > 80% of female patients and is an important concern for patients and their parents. Healthcare providers are often consulted for subfertility treatment possibilities. An option brought up by the families is intrafamilial oocyte donation (mother-to-daughter or sister-to-sister). In addition to POI, galactosemia patients can also present varying cognitive and neurological impairments, which may not be fully clear at the time when mother-to-daughter oocyte donation is considered. Ethical and societal aspects arise when exploring this option. This study aimed to provide guidance in aspects to consider based on the views of different groups involved in the oocyte donation process. A qualitative study using in-depth semi-structured interviews with > 50 participants (patients, family members, and healthcare providers) was conducted. From these interviews, themes of concern emerged, which are illustrated and reviewed: (1) family relations, (2) medical impact, (3) patients’ cognitive level, (4) agreements to be made in advance and organization of counseling, (5) disclosure to the child, and (6) need for follow-up. We conclude that discussing and carrying out intrafamilial oocyte donation in galactosemia patients requires carefully addressing these themes. This study adds value to the already existing recommendations on intrafamilial oocyte donation in general, since it highlights important additional aspects from the perspectives of patients and their families.

**Electronic supplementary material:**

The online version of this article (10.1007/s10545-018-0179-y) contains supplementary material, which is available to authorized users.

## Introduction

Primary ovarian insufficiency (POI) with subfertility is an important concern in classic galactosemia patients and their families. With a prevalence of > 80% in female patients, POI is the most common complication, representing the greatest psychological burden for women with this disorder (Kaufman et al. 1986; Bosch et al. 2004; Fridovich-Keil et al. 2011). Classic galactosemia is a rare inherited disorder of galactose metabolism caused by severe deficiency of the second step in the main pathway for galactose catalyzed by galactose-1-phosphate uridylyltransferase (GALT) (Coelho et al. 2017). Although a galactose-restricted diet quickly relieves the initial severe illness, diet-independent complications occur later in life, including POI. Female patients present a wide phenotypic spectrum of POI that can vary from primary amenorrhea to normal pubertal development, but irregular or absent menstrual cycles at a later stage (Rubio-Gozalbo et al. 2010; Fridovich-Keil et al. 2011). Although spontaneous pregnancies occur despite the presence of POI (Gubbels et al. 2008; van Erven et al. 2017), concerns about subfertility remain. Healthcare providers are frequently consulted for subfertility treatment possibilities. An option that is often brought up by families is intrafamilial oocyte donation, from mother to daughter (intergenerational) or from sister to sister (intragenerational). However, an important aspect in this disorder is a varying spectrum in cognitive impairments, including intelligence quotient (IQ, 47–122), in addition to POI (Waisbren et al. 2012; Welling et al. 2017). When mother-to-daughter oocyte donation is considered, this cognitive impairment may not yet be clear, in view of the young age of the galactosemic daughter. Intrafamilial oocyte donation raises ethical and societal questions, and professionals might be reluctant to mention this option. Several documents have recently been published on intrafamilial oocyte donation that address some of the concerns raised by this procedure (Nederlandse Vereniging voor Obstetrie en Gynaecologie, NVOG 2016; Balkenende et al. 2017).

This study aimed to provide guidance in aspects to consider when discussing this option in this specific group, based on the views of different groups involved in the oocyte donation process, including patients and their families.

## Methods

### Study design and selection of participants

This qualitative study was conducted in the Maastricht University Medical Center+, the Netherlands. The aim of this study was to evaluate the views and attitudes regarding ethical and societal aspects of intrafamilial oocyte donation in women with classic galactosemia. Possible participants were informed about this study in person, at the outpatient’s clinic, or by telephone when consent to be re-contacted was given in previous studies. When participants were interested, written information was sent. Patients (≥ 18 years of age), family members, and healthcare providers with different specialties involved in the treatment or follow-up of galactosemia patients were solicited for this study. This allowed us to collect a wide range of opinions, from different perspectives. Participants were enrolled from January 2017 to March 2017.

Approval for this study from our Research Ethics Committee was requested. After reviewing the study protocol, the committee agreed that official review and approval of this study was not necessary, due to the fact that it did not involve medical information. Written informed consent was obtained from all patients to participate in the study.

### Questionnaire and data collection

In-depth interviews using a semi-structured questionnaire were conducted, adjusted for the different groups (example in the supplementary material [English version]). The first author developed the questionnaire after examination of the literature and day-to-day experience with patients and other care providers, and discussed the questions with the other authors. When consensus was reached, the final version was drafted. Both close-ended and open questions were used to ensure that the questions would capture the relevant themes concerning intrafamilial oocyte donation. Through open-ended questions, participants were free to explore their views on different topics. Interviews with 14 patients, 19 family members, and 34 professionals from four academic centers in the Netherlands were conducted, between April 2017 and July 2017 (the characteristics of the participants are available in the supplementary material). All interviews were conducted by the first two authors. Interviews lasted between 30 and 45 min and took place over telephone or in the working place of the respondent. All interviews were audiotaped and transcribed verbatim. When no new information was derived from the interviews, saturation was reached, and participant enrolment was stopped (Guest et al. 2006).

### Data analysis

Data analysis was performed based on the constant comparative method (Malterud 2001). Views from different groups were collected and compared. The first two authors identified the texts that reflected the relevant themes emerging from the interviews. In consultation with the last author, the topics were refined and further developed. To highlight the different topics, representative quotations were identified.

## Results

Intrafamilial oocyte donation comes with many delicate issues. One of the interviewed professionals summarized it very well: “*I must admit this type of donation has some difficult sides to it, but those are definitely not insurmountable*.”

Six main themes to consider when discussing this option were identified through these interviews: (1) family relations, (2) medical impact, (3) patient’s cognitive level, (4) agreements to be made in advance and organization of counseling, (5) disclosure to the child, and (6) need for follow-up.

### Theme 1: can intrafamilial oocyte donation put family relations at risk?

Most family members and professionals express their concern that overattachment of the donor to the offspring and different opinions in raising the child could cause role conflicts, as illustrated by the quotation below.

Donating oocytes can lead to a different view of the child; you will get a grandchild and a child at the same time. For both parties, this would be a psychological burden. You must be aware that despite they are your oocytes; you are not really the child’s mother. In some cases, this could lead to difficult situations.

However, the psychologists we interviewed state that this should not be a problem if families openly inform the child about the oocyte donation.

Remarkably, possible role confusion created by intrafamilial oocyte donation seems not to be a point of concern in patients. It seems that a low education level is associated with less concerns regarding role conflicts. In this study, 3/14 patients were in a relationship (see the supplementary material). One of the partners agreed on participation in this study. The participant’s view was that their partner’s opinion should also be explored.

#### Intra- vs. interfamilial oocyte donation

Most participants agree that conflicts can arise in intragenerational oocyte donation, when the sister has not fulfilled her own wish for children before donating oocytes. Accordingly, sisters who proceeded in the process and were considering oocyte donation for their galactosemic sister emphasize on the need for psychological counseling. Also, participants foresee a higher chance of unsolicited interference with the child’s nurture in intergenerational donation, based on the family hierarchy, thereby, possibly having more impact on family relations.

#### Pressure on the donor and recipient

On one hand, intrafamilial oocyte donation could lead to pressure on the donor. The mothers we interviewed mentioned the possibility of donating oocytes out of a moral obligation, since it is a way to help their child. However, according to ethicists, acting from perceived moral obligation does not mean that donating oocytes is somehow less voluntary. They state that, with proper counseling, it is the competent donor’s choice to judge the proportionality of the treatment, since the respect for reproductive autonomy applies.

On the other hand, transgenerational pressure can occur, as one of the ethicists explains:

The notion that your mother has gone through this process with hormone stimulation and donation, might give the daughters a feeling of guilt.

Furthermore, a patient’s future reproductive autonomy can, thereby, be undermined. However, patients did not express feeling obliged to use the donated oocytes, even when knowing the effort that has been made. Identity problems in the offspring are also mentioned as a point of concern by professionals.

Regardless of the possible effects on family relations, participants view this technique as something beautiful, if considered carefully. The perceived greatest advantage of intrafamilial oocyte donation is genetic closeness, mentioned by all participants.

### Theme 2: which medical aspects should be considered?

All participants agree that considering the physical condition of the donor and recipient, including medical background, is important. Additionally, professionals emphasize consideration of the proportionality of intrafamilial oocyte donation. This includes the burdens and risks (infertility due to bleeding/infection) of the IVF treatment on one side and the possible benefits on the other side. Thereby, a mother’s or sister’s efforts could be in vain:

The galactosemia patient might get pregnant spontaneously, not want children, not have a partner, decide to adopt, find another donor or be so impaired that having children is not an option.

### Theme 3: patient’s cognitive level

Participants agree that cognitive level must be considered when evaluating the support system for a child.

We do not need perfect parents with a high education; it is about love, cordiality and providing a safe environment for the child to grow up in.

However, some professionals wonder whether some of these patients have the cognitive capacity to oversee the process of intrafamilial oocyte donation. Another concern mentioned by professionals is the possibility of assortative mating. Patients who might have cognitive impairments are likely to be in a relationship with somebody with a similar phenotype.

### Theme 4: agreements to be made in advance and organization of counseling

Professionals point out that clear agreement on ownership of the oocytes should be made. Counseling the oocyte recipient must be done according to existing guidelines (NVOG 2010, 2016):

A multidisciplinary team should evaluate the whole system around a candidate: is there a stable and safe environment for the child and how does the social network look like?

This multidisciplinary approach should include a consultant in metabolic diseases, gynecologist, psychologist, social worker, and others, if needed.

### Theme 5: disclosure to the child

Quote from a psychologist:

Secrets cost a lot of energy and the chance of the secret unintentionally being revealed at some point is high. The trick is to make it normal for the child; you can achieve this by informing him/her from a young age, in an understandable way.

Psychologists state that openly informing the offspring about the oocyte donation at a young age should be encouraged. However, there are different views on disclosure amongst other participants. Most of them would openly inform the offspring about the method of their conception. An age when the child can understand the situation is preferred.

### Theme 6: need for follow-up of the offspring?

Recommendations regarding follow-up of the offspring are missing in the current guidelines. Some professionals think it is preferable to have a standard check-up occasionally. Others think you should not medicalize the oocyte donation and not offer standard care but keep the possibilities for medical and psychological support easily accessible.

## Discussion

Our results show that intrafamilial oocyte donation in this group of patients comes with different considerations that go beyond the existing recommendations.

### Theme 1: intrafamilial oocyte donation and family relations

Motives and expectations regarding involvement in raising the child and social roles should be evaluated when counseling patients and their family members. Donors, recipients, and partners need counseling together, but also apart from each other. Furthermore, it is important to evaluate voluntariness to donate/use the donated oocytes. There are several studies on intrafamilial oocyte donation in general that evaluated the effect on family relations and the psychological well-being of the child. Intragenerational oocyte donation in the general population does not necessarily have a negative impact on family relations. Social rather than genetic connection seems to be of importance in the nature of the relationship with the child (Jadva et al. 2011; Ilioi et al. 2017). Family relations, therefore, do not seem to be affected. Furthermore, there is a gestational and genetic connection between the mother and the child, suggesting fewer adjustment problems in the offspring (Golombok et al. 2013). The possible effect on the identity of the child has been evaluated in father-to-son sperm donation. Despite it possibly being emotionally disturbing for the child and (negatively) affect the child’s identity, it also allows the child to know its genetic background and be close to the biological parent (Bredenoord et al. 2012). Studies do not show differences in psychological adjustment between children born through gamete donation and naturally conceived children (Golombok et al. 2011, 2013). Although some participants expressed their concern, data from the literature so far suggest no psychological problems in the offspring.

After reviewing the literature, we found two reports of intrafamilial oocyte donation in other genetic disorders. The first report describes the cryopreservation of a mother’s oocytes for possible future use by her daughter with Turner syndrome. The main outcome of this report is the number of cryopreserved oocytes. Possible limitations of this procedure are mentioned, including problems with family relations (Gidoni et al. 2008). The second report describes a sister-to-sister oocyte donation, where both sisters have intermediate-size mutations in fragile X mental retardation, with an increased risk for POI (Rybak et al. 2009). This report revealed that both sisters preferred sibling donation over anonymous oocyte donation, despite possible problems in family relations.

#### Inter- vs. intragenerational oocyte donation

Several differences were mentioned. First, fresh oocyte donation is possible in intragenerational oocyte donation, whereas there is a need for cryopreservation of the donated oocytes for a possible future need in intergenerational oocyte donation. In view of the patient’s cognitive level, if cognitive impairment occurs in patients, this will be fully clear at time when intragenerational oocyte donation is considered, as opposed to the intergenerational oocyte donation. This means that the donation and corresponding emotional stress the donor has gone through could be in vain. Since a sister can donate fresh oocytes when needed, the ethical concerns regarding pressure from third parties to use the oocytes are less likely to play a role. The literature shows that the majority of patients with POI would prefer a sister donating oocytes instead of anonymous oocyte donation (Sauer et al. 1988; Lessor et al. 1990; Ethics Committee, American Society for Reproductive Medicine, ASRM 2003).

The European Society of Human Reproduction and Embryology (ESHRE) points out that genetic closeness is a generally accepted reason for investing time and resources into treatment (ESHRE Task Force on Ethics and Law et al. 2011). A mother or sister would be a healthy, easily accessible source of oocytes, according to professionals. This is important because, in general, women who suffer from POI are required to find an oocyte donor among their own acquaintances, as oocyte donors are rare. Also, the process is described as less stressful when the donor is known to the recipient (ASRM 2003).

### Theme 2: medical aspects to consider

When counseling patients, it is important to mention that the success rate of this fertility treatment is limited. Furthermore, to guarantee an appropriate number of oocytes, several attempts could be required. Since there is a higher chance that the child may be a genetic carrier of galactosemia, screening the patient’s partner could be considered.

### Theme 3: patient’s cognitive level

As mentioned before, cognitive impairment can occur in classic galactosemia patients. One must bear in mind that possible cognitive impairments may not be clear at the time intergenerational oocyte donation is discussed. Proper and understandable information regarding the procedure and possible psychological consequences is required. It is very important to evaluate the patient’s social network and environment even more carefully, especially in the long term, so that potential needs can be acknowledged and necessary measures arranged.

### Theme 4: agreements to be made in advance and organization of counseling

It is important to agree on the ownership of the oocytes according to the law in the different countries. European legislation in the field of medically assisted reproduction often differs between countries and not all European countries have specific legislation (Busardò et al. 2014). According to Dutch embryo law (http://wetten.overheid.nl/BWBR0013797/2013-09-27), the oocytes stay the property of the donor until they are used by the acceptor. Hereby, mothers could possibly put terms and conditions upon the donation. Considering the dependency of the daughter on the mother’s will to donate oocytes, this could create an unequal relationship and the oocytes can potentially be used for bribery. Therefore, it would be preferable for the daughter to have the right to use the oocytes from her 18th birthday (Balkenende et al. 2017).

### Theme 5: disclosure to the child

The results of our study show that early disclosure to the child is important. This confirms findings in earlier studies. A study by Golombok et al. has shown that children born through assisted reproduction, in general, may benefit from disclosure about the nature of their conception before they enter school (Golombok et al. 2011). The quality of family relationships and the psychological well-being of children born through gamete donation would be better if they are told about the nature of their conception (Ilioi et al. 2017). Evidence that keeping the method of conception a secret from the child would cause harm is minor. However, the chance of unintentional disclosure is expected to be high, especially in intrafamilial donation, which may be harmful for the child. Counseling regarding disclosure is, therefore, recommended (ESHRE 2011). Another argument for disclosure that has been mentioned is the right for the child to know their biological origins, for both psychological and medical reasons. This decision should be made by the parents.

### Theme 6: need for follow-up

The galactosemia patients are seen in the outpatient clinic annually. Problems regarding social situation and role confusion could be discussed, if necessary. During the counseling for this procedure, the option of seeking professional help for the child, if needed, must be discussed.

This study was conducted in the Netherlands. However, we do think that the findings in our study are representative of other European/western countries and could be used as a guideline when being confronted with questions regarding intrafamilial oocyte donation. Most studies evaluating the views on oocyte donation in general derive from the USA or the UK, and our results are in line with the findings in those studies (Purewal and van den Akker 2009; ASRM 2012). Also, the ESHRE (2011) has provided different recommendations regarding ethical aspects in intrafamilial oocyte donation that are considered applicable to all European countries.

## Conclusion and considerations for counseling

We conclude that intrafamilial oocyte donation in this group of patients comes with different considerations. The topics to be considered when discussing intrafamilial oocyte donation for galactosemia patients are family relations, medical impact, patient’s cognitive level, agreements to be made in advance and organization of counseling, disclosure to the child, and need for follow-up, as summarized in Table 1.

**Table 1:** Considerations for counseling intrafamilial oocyte donation in galactosemia patients

Family relations
- Evaluate expectations of different parties: donor, recipient, and partner, regarding involvement in child raising and disclosure. - Evaluate motives to donate/use oocytes. Ensure that this is done voluntary and thought through carefully. - Evaluate if donor has fulfilled own wish to have children. - Mention that no rights can be derived from intrafamilial oocyte donation.
Medical impact
- Discuss proportionality of the IVF treatment; possible burdens, risks, and benefits of the donation process, including the success rate of IVF treatment. - Evaluate physical condition of donor and recipient and medical background. Consider testing donors and patients’ partners as possible carriers of galactosemia.
Cognitive level
- Evaluation of patients’ social environment and network by a psychologist/social worker. - Consider fertile age of the mother; inform the parents of galactosemia patient as soon as possible but explain that intrafamilial oocyte donation is an option that her daughter might never (want to/could) use. - Provide proper information regarding the procedure and possible psychological consequences. - Additional support may be considered.
Agreements to be made in advance and organization of counseling
- Counseling and evaluating of the potential oocyte acceptor by a multidisciplinary team, according to existing recommendations and moral fertility treatment contra-indications protocol. - Consider when to inform the galactosemia patients in case of intergenerational oocyte donation.
Disclosure to the child
- The ultimate decision about disclosure is made by the parents. - Counseling regarding disclosure and possibility of unintentionally revealing the method of conception is recommended. - If disclosure is preferred, advise families to inform the offspring at a pre-school age.
Follow-up
- Evaluate social environment and potential role confusion routinely. This can be done by the involved internist in the annual outpatient check-up, but it might be preferable to let a psychologist carry out the follow-up, since there is no experience in this patient population yet. - Keep in mind that some patients might be lost to follow-up. - No standard psychological follow-up of the child to be born is considered necessary.

## Electronic supplementary material

Below is the link to the electronic supplementary material.

ESM 1 (DOCX 137 kb)
