# Supplementary material for: Intrafamilial oocyte donation in classic galactosemia: ethical and societal aspects
Source: J Inherit Metab Dis. 2018 Apr 18;41(5):791–7. doi: 10.1007/s10545-018-0179-y (PMC6133175; doi:10.1007/s10545-018-0179-y)
Supplement: Supplementary file 1 — (DOCX 137 kb) [file 10545_2018_179_MOESM1_ESM.docx]

SUPPLEMENTAL APPENDIX 1: Patients’ questionnaire


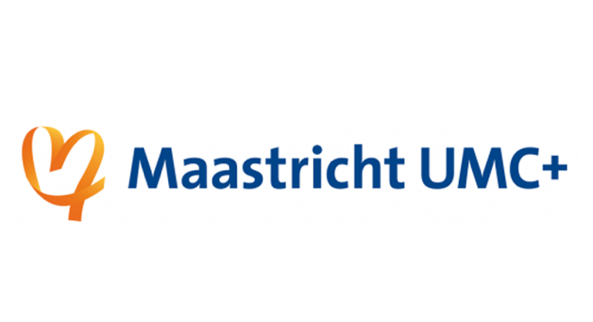


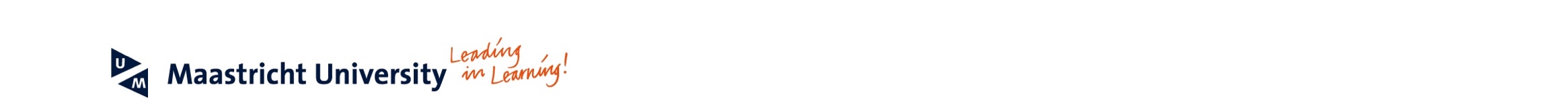


**Patients’ questionnaire: 'intra-familial oocyte donation in classical galactosemia: ethical and social impact'**

This questionnaire will be used in the interview (by telephone or in person) that you will have with the researcher and is sent to you in advance to be prepared for the questions. You do not need to fill in this list, but because you may want to think about a few things, we would like to ask you to read this carefully.

If you have any questions, please contact the researcher, Minela Haskovic (m.haskovic@student.maastrichtuniversity.nl). You can also ask your questions during the interview.

**General information**

1. What is your age? ……… years

2. What is your profession? …………………………………………

3. What is your highest level of education? ........................................................................

- What is your highest level of your father? …………………………………......................................................................................

- What is your highest level of your mother? …………………………………......................................................................................

- What is your highest level of your siblings?

…………………………………………………………………………………………..

**Classic galactosemia**

4. At what age is classis galactosemia diagnosed?

..........................................................................................................................................

5. Which part of the disease causes the most trouble/inconvenience in your daily life?

…………………………………………………………………………………………..

6. Are you familiar with primary ovarian insufficiency (POI)? **No/Yes**

Which problems could you experience due to the POI? …………………………………………………………………………………………..

Do you think there is something we can do about it? If yes, explain what

…………………………………………………………………………………………..

7. Do you have (biological) children?

How many? ……………………………………………………………………………..

If no, have you ever considered children?

Elaborate on your answer? ..........................................................................................................................................

8. What are other options to conceive, when natural conception fails?

…………………………………………………………………………………………..

9. Have you ever heard of intra-familial oocyte donation (mother-to-daughter or sister-to-sister?

If yes, what is your opinion on this?

.………………………………………………………………………………………….

10. What are the pros and cons of intra-familial oocyte donation? When

compared to non-familial oocyte donation?

..........................................................................................................................................

11. Are there differences between mother-to-daughter and sister-to-sister donation? Explain your answer

..........................................................................................................................................

12. What are aspects to consider when considering intra-familial oocyte donation in classic galactosemia patients?

..........................................................................................................................................

How should this be organized?

..........................................................................................................................................

13. Would you feel obligated to use the donated oocytes?

…………………………………………………………………………………………..

14. How would this process affect the relationship between the recipient (galactosemia patient) and donor (mother/sister)?

…………………………………………………………………………………………

15. How would this procedure influence each person’s relationship with the child?

..........................................................................................................................................

15. How should roles in raising the child be organized?

..........................................................................................................................................

16 Would u openly inform the child about the way of conception?

Explain your answer

..........................................................................................................................................

17. What are possible effects of intra-familial oocyte donation on the child? Is psychological support for the child desirable? How should this be organized?
……………………………………………………………………………………….....

SUPPLEMENTAL APPENDIX 2: Characteristics of Participants

*Supplemental table 2. Characteristics interviewed professionals*

| **Professional** | **Department** | **Profession** |
| --- | --- | --- |
| 1 | Clinical Genetics/ Genetics and Cell Biology | Laboratory Specialist |
| 2 | Clinical Genetics/ Genetics and Cell Biology | Researcher |
| 3 | Clinical Genetics/ Genetics and Cell Biology | Laboratory Specialist |
| 4 | Clinical Genetics/ Genetics and Cell Biology | Laboratory Specialist |
| 5 | Pediatrics | Researcher |
| 6 | Clinical Genetics/ Genetics and Cell Biology | Analyst |
| 7 | Clinical Genetics/ Genetics and Cell Biology | Laboratory Specialist |
| 8 | Clinical Genetics/ Genetics and Cell Biology | Researcher |
| 9 | Clinical Genomics | Researcher |
| 10 | Rehabilitation | Researcher |
| 11 | Dietetics | Dietitian |
| 12 | Internal medicine | Internist |
| 13 | Internal medicine | Internist |
| 14 | Pediatrics | Pediatrician  Metabolic Diseases |
| 15 | Pediatrics | Pediatrician  Metabolic Diseases |
| 16 | Pediatrics | Pediatrician  Metabolic Diseases |
| 17 | Pediatrics | Pediatrician  Metabolic Diseases |
| 18 | Medical ethics | Ethicist |
| 19 | Medical ethics | Ethicist |
| 20 | Medical ethics | Researcher |
| 21 | Medical ethics | Ethicist |
| 22 | Reproductive medicine | Gynecologist |
| 23 | Reproductive medicine | Gynecologist |
| 24 | Reproductive medicine | Gynecologist |
| 25 | Clinical Genetics | Clinical geneticist |
| 26 | Psychology | Child psychologist |
| 27 | Reproductive medicine | Gynecologist |
| 28 | Psychology | Psychologist/Infant mental health specialist |
| 29 | Internal medicine | Internist |
| 30 | Pediatrics | Pediatrician  Metabolic Diseases |
| 31 | Reproductive medicine | Gynecologist |
| 32 | Psychology | Psychologist |
| 33 | Medical ethics | Ethicist |
| 34 | Internal medicine | Internist |

*Supplemental table 3. Characteristics interviewed patients*

| Patient | Age | Education level (ISCED 2011) | Partner (Y/N) |
| --- | --- | --- | --- |
| 1 | 19 | 3 | N |
| 2 | 23 | 2 | N |
| 3 | 26 | 2 | Y |
| 4 | 23 | 4 | Y |
| 5 | 20 | 3 | N |
| 6 | 23 | 2 | N |
| 7 | 28 | 3 | Y |
| 8 | 20 | 2 | N |
| 9 | 21 | 6 | N |
| 10 | 20 | 6 | N |
| 11 | 50 | 6 | N |
| 12 | 33 | 3 | N |
| 13 | 19 | 3 | N |
| 14 | 21 | 2 | N |

*Supplemental table 4. Characteristics interviewed family members*

| Family member | Age | Education level (ISCED 2011) |
| --- | --- | --- |
| 1 | 51 | 3 |
| 2 | 49 | 3 |
| 3 | 53 | 6 |
| 4 | 57 | 4 |
| 5 | 49 | 4 |
| 6 | 56 | 3 |
| 7 | 49 | 3 |
| 8 | 52 | 6 |
| 9 | 58 | 6 |
| 10 | 47 | 4 |
| 11 | 41 | 3 |
| 12 | 51 | 2 |
| 13 | 28 | 6 |
| 14 | 26 | 3 |
| 15 | 24 | 6 |
| 16 | 26 | 3 |
| 17 | 30 | 6 |
| 18 | 47 | 6 |
